# Kidney-Sparing Management of UTUC in Solitary Kidney Patients: A Retrospective Analysis and Narrative Review

**DOI:** 10.3390/jcm13226788

**Published:** 2024-11-11

**Authors:** Angelis Peteinaris, Spyridon Polyzonis, Vasileios Tatanis, Theodoros Spinos, Paraskevi Katsakiori, Theofanis Vrettos, Evangelos Liatsikos, Panagiotis Kallidonis

**Affiliations:** 1 Department of Urology, University Hospital of Patras, 26504 Patras, Greece; spyrpolyzonis@gmail.com (S.P.); tatanisbas@gmail.com (V.T.); thspinos@otenet.gr (T.S.); vkatsak@gmail.com (P.K.); liatsikos@yahoo.com (E.L.); pkallidonis@yahoo.com (P.K.); 2 Department of Anesthesiology and ICU, University Hospital of Patras, 26504 Patras, Greece; teovret@gmail.com; 3 Department of Urology, Medical University of Vienna, 1090 Vienna, Austria

**Keywords:** upper tract urothelial carcinoma, endoscopic management, high-grade UTUC

## Abstract

**Background/Objectives:** The aim of this study is the presentation of an endoscopic therapeutic approach for three patients with a solitary kidney who were diagnosed with urothelial cancer of the upper tract. **Methods:** This retrospective analysis included patients with solitary kidneys who suffered from high-grade UTUC (urothelial cancer of the upper urinary tract) and underwent conservative treatment. **Results:** The first patient was a 67-year-old male who had a prior history of a nephroureterectomy due to UTUC six years ago. The patient was diagnosed with high-grade UTUC in the contralateral kidney. The tumor has been managed with endoscopic ablation. The second patient was a 74-year-old male with a non-functional kidney and high-grade UTUC diagnosed in the contralateral side. The patient underwent endoscopic ablation for the tumor. The third case was a 68-year-old female patient who had a history of a nephroureterectomy due to UTUC. Afterward, she was diagnosed with high-grade UTUC in the contralateral kidney. The patient was treated with percutaneous tumor resection and the placement of a nephrostomy tube. The first patient was included in an immunotherapy program based on an oncologist consultation after laser ablation treatment for Ta high-grade UTUC, followed by the endoscopic management of two recurrences. Afterward, no recurrence was detected. The remaining two patients followed up without the detection of a new recurrence. **Conclusions:** The kidney-sparing approach (tumor laser ablation or resection) for high-risk UTUC treatment in selected patients with solitary kidneys seems to provide adequate early outcomes in relation to preserving renal function and effective disease management. It is important to personalize the way of treatment in every case after a thorough examination of the patient’s data.

## 1. Introduction

Urothelial carcinoma (UC) is the second most prevalent urological cancer in developed countries [1]. UCs can be localized either in the lower (bladder and urethra) and/or the upper urinary tract (pyelocalyceal cavities and ureter) [2]. Upper tract urothelial carcinoma (UTUC) is not so common compared to bladder cancer, representing 5% of urothelial cancer cases, while bladder cancer approaches 95% [3]. Nevertheless, the incidence rates have been rising as diagnostic evaluation has evolved and due to the increasing life span of the human population. Furthermore, the greater quality of imaging and the addition of flexible ureteroscopes in the diagnostic armamentarium also play important roles. This has resulted in a higher diagnosis rate in elder patients, creating a growing demand for minimally invasive approaches that combine treatment and renal function preservation [4]. Over the past decade, the survival rates for UTUC have seen minimal improvements [5]. The location of UTUC complicates both diagnosis and staging [5]. Approximately 25% of upper tract cancers originate in the ureteral lumen. The rest (75%) develop in the pelvis, infundibula and calyces [5]. UTUC appears more often in males, mostly between 70 and 90 years of age [6]. More than half of the patients are diagnosed after symptoms have occurred, while smoking is one of the strongest risk factors [7]. Aristolochic acid, present in numerous Chinese herbal medicines, was found to be linked to urothelial TP53 mutations, leading to a higher incidence of UTUC in specific regions [5]. Although visible hematuria constitutes the most common symptom, only 0.75% of patients with macroscopic hematuria are diagnosed with UTUC and the percentage further decreases to 0.17% in the case of microscopic hematuria [3]. In addition, there is a strong association between Lynch syndrome as a genetic risk factor and UTUC [8]. A hereditary predisposition to urothelial cancer of the upper tract accounts for 10–20% of all cases. MMR (mismatch repair genes) alterations are strongly linked to hereditary non-polyposis colorectal carcinoma (HNPCC) or even the aforementioned Lynch syndrome [5]. A ureteroscopy (URS) is considered one of the primary diagnostic tools. This approach allows the surgeon to receive samples for cytology and also retrieve a biopsy of macroscopically suspected lesions. Nevertheless, obtaining tissue samples via the ureteroscope is a technically challenging procedure and discordance between the biopsy results and final pathology findings is not a rare phenomenon [5].

UTUC is variable and it ranges from low-grade, indolent lesions to aggressive, high-grade tumors that have a high risk of metastasis. This aggressive behavior often necessitates early and radical treatment. The gold standard treatment for UTUC is a radical nephroureterectomy (RNU) with a bladder cuff excision, regardless of the tumor location [9]. Nevertheless, the increased possibility of complications, such as the deterioration of renal function, which might impact oncological management, has increased the popularity of nephron-sparing approaches. Consequently, various treatment approaches for low-grade UTUC have been explored and reported [10]. However, kidney-sparing techniques, such as endoscopic ablation and a segmental urethrectomy (SU), are ever more considerable options in selected patients [11]. Regarding the latest guidelines for UTUC management, a kidney-sparing approach may be applied to all cases of localized, low-risk tumors, irrespective of the status of the contralateral kidney [11].

Low-risk UTUC is defined as unifocal, low-grade lesions with a diameter of less than 2 cm without any invasive marks on a computed tomography (CT) scan. At the same time, both the selective and voided urine cytology should be classified as low-grade. In these cases, laser ablation via ureterorenoscopy or percutaneous access, segmental ureterectomy or even chemo-ablation could be available choices of treatment, considering the characteristics of each individual [12]. As far as high-risk UTUC is concerned, it is characterized by tumors larger than 2 cm, hydronephrosis of the ipsilateral kidney, high-grade disease found on a cytologic report or a biopsy, histological variants of urothelial cancer, multifocal disease, or a history of bladder cancer treatment [11]. The standard approach is considered to be an open or minimally invasive radical nephroureterectomy (RNU) with a bladder cuff excision. The surgical advancements of the last decades have decreased perioperative morbidity, but UTUC survival rates have remained stagnant for almost 20 years, underlining the importance of improved treatment strategies [5]. Therefore, high-risk UTUC kidney-sparing management could be considered in selected cases such as a bilateral tumor, renal insufficiency, solitary kidney or in patients unfit for a radical nephroureterectomy [11]. Thus, the treatment approach for each patient should be selected after a thorough discussion of all the available treatment options and possible outcomes [13]. Considering the necessity of the preservation of renal function, patients with a solitary kidney may be candidates for conservative treatment for UTUC.

This study aims to present the treatment and surgical approaches for three patients with solitary kidneys who were diagnosed with high-grade urothelial cancer of the upper urinary tract. In all cases, conservative management was performed, using laser ablation and percutaneous approaches in order to preserve renal function.

## 2. Materials and Methods

In this case series, we retrospectively analyzed the data of patients with solitary kidneys who suffered from high-grade UTUC and underwent conservative treatment between January 2016 and May 2024. The presence of one functional kidney and the diagnosis of high-grade upper tract urothelial cancer were considered to be the inclusion criteria. We retrospectively analyzed the information from the database of the Department of Urology at the University Hospital of Patras in order to find patients that match the aforementioned criteria. The lack of data, loss from follow-up or the application of radical nephroureterectomy were considered exclusion criteria. The oncological outcomes and survival were the main recorded endpoints. In total, three patients were included in this study.

## 3. Results

**Figure 1:**
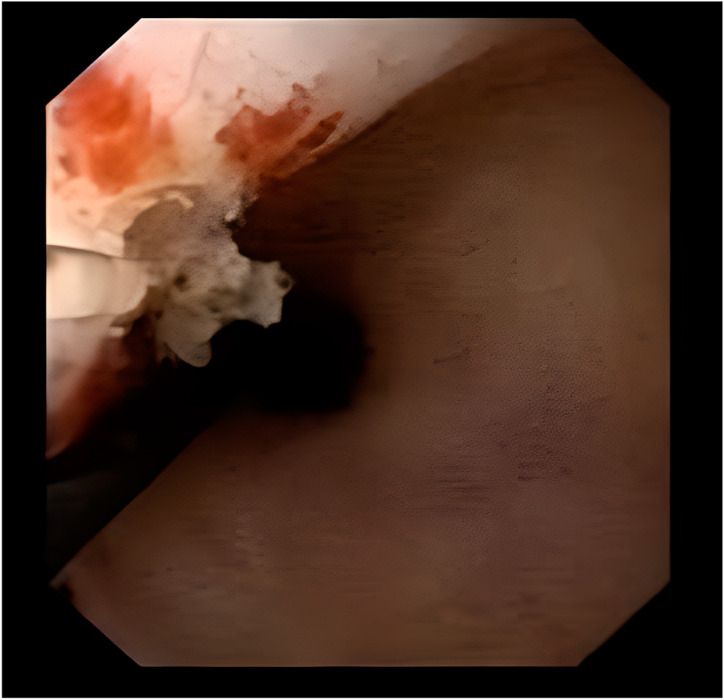
Endoscopic ablation of the high-grade tumor of the first patient.

The first patient was a 67-year-old male who underwent a right radical nephroureterectomy due to multifocal UTUC on the right kidney. The pathological report detected pT3 stage UTUC in the renal pelvis and pT1 stage UTUC in the upper ureter. The patient had a history of well-regulated hypertension, diabetes and almost 40 pack-smoking years, although he had ceased smoking after the first surgery. During follow-up, the patient was diagnosed with low-grade bladder cancer and underwent multiple transurethral resections. One year ago, the patient presented with macroscopic hematuria. He underwent a cystoscopic evaluation, while cytology and a computed tomography urogram were also conducted. The urine cytology was negative, but the presence of a contrast defect observed during the urogram raised suspicion for the presence of UTUC. Urothelial carcinoma in the renal pelvis of the solitary kidney was detected during the diagnostic ureterorenoscopy (Figure 1). The biopsies, which were extracted by forceps, revealed Ta high-grade urothelial cancer and after a thorough discussion with the patient, ureterorenoscopic laser ablation of the tumor constituted the treatment of choice.

The second patient was a 74-year-old male with a medical history of Ta low-grade bladder cancer and a non-functional right kidney due to recurrent nephrolithiasis (5% split function of the corresponding kidney was revealed during the most recent DMSA renal scan). Regarding the personal history, the patient has well-regulated diabetes and hypertension. In addition, the patient had a history of coronary heart disease, atrial fibrillation and hyperuricemia. Two years ago, after some episodes of spontaneous gross, painless hematuria, which was self-regulated, the patient visited the outpatient unit of our Department. Despite the history of urolithiasis, the patient was also a heavy smoker, so the work-up included urine cytology, a computed tomography urogram and also a cystoscopy. The cytology was once again negative, while contrast uptake was reported in the middle and lower calyceal groups of the functional left kidney. After discussing the work-up with the patient, an ureterorenoscopy was decided on as the course of treatment. Biopsies were taken during the surgery, and he was diagnosed with Ta high-grade multifocal urothelial cancer in the middle and lower calyces of the left functional kidney. A ureterorenoscopic laser ablation of the tumor followed.

**Figure 2:**
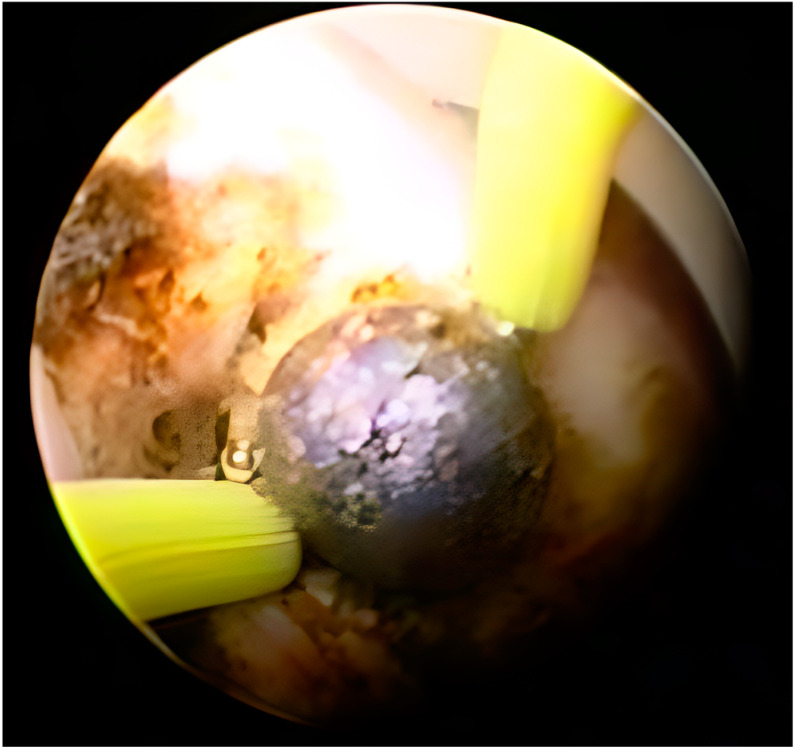
Percutaneous resection of the high-grade tumor of the third patient.

The third case was a 68-year-old female patient with a medical history of a radical nephroureterectomy due to T1 high-grade pelvic urothelial cancer, about 25 years ago. No comorbidities were present from the history of the patient. Over the past two decades, she has been diagnosed with Ta high-grade bladder cancer that was transurethrally resected and well managed with BCG instillations. About five years ago, the patient presented in the emergency unit of the hospital, reporting a gradual change in the color of her urine, and thinking that there was probably a bladder recurrence. The cystoscopic evaluation did not reveal a bladder tumor, and the patient underwent a computed tomography urogram, which presented the existence of a big mass in the pelvis of the remaining kidney. An ureterorenoscopy was conducted and the patient was officially diagnosed with UTUC in the contralateral kidney. The tumor was >2 cm in size and the biopsy revealed it was at the T1 stage. After some weeks, the patient presented with flank pain due to an ureteropelvic junction obstruction caused by the tumor. After discussing it with the patient and taking into consideration that retrograde access was not possible, percutaneous access was used, followed by tumor resection and a nephrostomy tube placement (Figure 2).

All the cases remained hospitalized for the first postoperative night, and they were discharged after foley catheter removal the next morning, after the intervention. There were no complications observed.

The pattern of follow-up, which was decided for all the patients, was the following:

All the patients would be followed up with a cystoscopy and urine cytology every 3 months for 2 years and every 6 months for 5 more years. A CT urography and chest CT would be performed every six months until the completion of two years and then yearly. In case of a possible recurrence, indicated by any of the aforementioned exams, a ureterorenoscopy would be considered in order to exclude the possibility of recurrence.

The first patient was included in an immunotherapy protocol based on his oncological consultation, after the laser ablation of the diagnosed Ta high-grade UTUC. During his follow-up, a positive cytology raised suspicion of a recurrence during the first two follow-up visits. An ureterorenoscopy was conducted in each case and a recurrence was detected in both the first and second diagnostic ureterorenoscopy. More precisely, recurrence in the middle calyx was detected during the 3-month diagnostic ureterorenoscopy that followed the positive cytology examination, while the 6-month diagnostic ureterorenoscopy revealed a recurrence in the lower calyx, again after a positive cytologic report. In both cases, laser ablation was performed. Since then, the patient has been followed up with for one year without a recurrence of the UTUC during diagnostic cystoscopic evaluations, selective and non-selective urine cytology or tumor suspicion after a CT urography. It is worth mentioning that despite the multiple surgeries, the patient’s creatinine levels have been stable and inside the range of normal, while during the last follow-up, it was found to be 0.9 mg/dL (eGFR 94 mL/min/1.73 m^2^).

The second patient has been followed up with for two years without suspicion of recurrence. The cystoscopies, the CT urography and the selective and non-selective urine cytology examinations have been negative until now. Nevertheless, a gradual increase in the patient’s creatinine levels has been observed. After the first nephroureterectomy, a baseline at the level of 1.4–1.6 mg/dL (eGFR 53–45 mL/min/1.73 m^2^) was maintained for years. The last two examinations revealed that the patient’s baseline has been altered to 1.8–2.1 mg/dL (eGFR 38–32 mL/min/1.73 m^2^) and the patient has also consulted with a nephrologist.

The third patient has been undergoing follow-ups for five years without the detection of tumor recurrence during the corresponding examinations. Due to postoperative stenosis of the ureteropelvic junction, the ureter remains stented, and the patient undergoes a JJ stent replacement every six months. The patient’s creatinine levels remain steady and within the normal range (0.9–1.1 mg/dL and eGFR 70–55 mL/min/1.73 m^2^). The removal of the JJ stent and a close follow-up were proposed to this participant, but she does not want to risk possible hydronephrosis and the deterioration of renal function. Nevertheless, the presence of this foreign body has led to four urinary tract infections that demanded hospitalization. The patient is currently receiving chemoprophylaxis as suggested after an Internal Medicine consultation.

## 4. Discussion

The outcomes of kidney-sparing management were evaluated in our study in patients with solitary kidneys diagnosed with high-grade UTUC. All three cases presented in this study underwent conservative treatment approaches, either with laser ablation or resection of the tumor. The treatment was achieved via a retrograde ureterorenoscopy or percutaneous access, followed by close and strict follow-ups. The histological evaluations revealed pure urothelial carcinomas for all three of the presented cases. Nevertheless, 1 out of 10 UTUC patients may present with a histological variant of urothelial carcinoma [14]. An association between variant histology and the possibility of metastatic disease and cancer-related death has been established, while overall survival and disease recurrence do not seem to be affected. Despite the aforementioned data, it is important to underline that the research for the management of histological variants is lacking strongly based scientific data, but also that these patients should be treated in an individualized, yet multidisciplinary manner [15].

Nearly two-thirds of patients with UTUC present with a high-grade disease. Due to the prevalence of high-grade cases, the lack of reliable data supporting the oncologic safety of conservative management and the limited validated criteria for selecting patients for endoscopic treatment, most UTUC patients require radical surgical resection [16]. High-risk UTUC is characterized by tumors larger than 2 cm, a dilated pelvicalyceal system, a high-grade tumor reported by a biopsy or cytology, urothelial histological variants, multifocal disease, or a history of bladder cancer treatment. The definite surgical approach for high-risk UTUC is more or less standardized, as the majority of the patients are guided to an open or laparoscopic radical nephroureterectomy with a bladder cuff excision [5]. Despite advances in surgical techniques, the survival rates for UTUC patients have remained unchanged over the years, underscoring the need for a more holistic approach for these patients [5].

Recent bibliography suggests that kidney-sparing alternatives have been gaining popularity in comparison to a radical nephroureterectomy and their implementation has become more and more frequent [10,17]. Despite the excellent oncological and functional results of minimally invasive techniques, a radical nephroureterectomy is a challenging operation, which demands surgical experience to be successful [18]. The management of the bladder cuff and the danger of the disease spreading are some of the milestones of minimally invasive approaches [19]. Nevertheless, clinicians should respect the indications of nephron-sparing approaches and keep the patients informed about the possible complications, and the need for strict follow-ups [17].

A radical nephroureterectomy can be accepted as the gold standard approach for cancers of the upper tract. Despite this, several concerns are expressed, mainly regarding the perioperative complications and the effect on renal function [10]. Several studies support that a radical nephroureterectomy can lead to complications in approximately 8–20% of the cases [10]. On the other hand, it is not rare for patients with UTUC to already have chronic kidney disease when they are diagnosed, while the deterioration of renal function is expected after a radical nephroureterectomy. It is well-established that chronic kidney disease is one of the possible complications for UTUC patients. Additionally, impaired renal function may make it challenging for patients to undergo future chemotherapy [10]. Therefore, the demand for nephron-sparing techniques has steadily increased, leading to the application of various treatment modalities in clinical practice.

Nephron-sparing surgery is currently considered a viable choice for selected patients, including patients with a solitary kidney, severe renal impairment and multiple bilateral UTUC [12]. Kidney-sparing surgical techniques include endoscopic management, like a ureteroscopic approach, percutaneous access and even a segmental ureterectomy. Percutaneous access is mainly utilized for tumors that are difficult to reach, such as lower pole tumors or tumors in patients with complex anatomy. The instillation of topical agents, including Bacillus Calmette-Guerin (BCG) or mitomycin C (MMC), can also be performed, combined with ureteroscopic or percutaneous access [12].

Endoscopic ablation during a ureteroscopy is among the most used kidney-sparing technique to treat UTUC. It is performed either via an antegrade or a retrograde approach, with the latter being used the most often [11]. Ablation techniques include tissue excision (by biopsy forceps or a basket), tumor resection (with the use of a resectoscope), electrocautery or laser ablation [20]. Endoscopic laser ablation was performed in two patients in our study, while the third patient underwent percutaneous access because retrograde access was impossible due to a ureteropelvic obstruction.

The primary aim of endoscopic surgery is the preservation of the kidney while maintaining optimal oncological results [11]. Although no prospective studies have compared the aforementioned treatments with a radical nephroureterectomy for UTUC, current data from retrospective studies suggest that both treatment approaches offer comparable oncological outcomes.

In patients with solitary kidneys, the preservation of renal function is paramount to avoiding dialysis and maintaining overall health. Kidney-sparing techniques, such as ureteroscopic laser ablation and a segmental ureterectomy, provide a significant advantage in retaining renal function, especially when compared to a radical nephroureterectomy, which inevitably leads to a decline in kidney function and associated risks like chronic kidney disease (CKD).

Fejkovic et al. compared 20 cases that underwent endoscopic ablation with 178 patients that underwent a radical nephroureterectomy (RNU), and reported that the ablation group presented superior postoperative kidney function [21]. However, it was found that the endoscopic treatment had a local recurrence rate of 61% for the ureteroscopic approach and 36% for the percutaneous approach, while the local recurrence after a radical nephroureterectomy is rare. The importance of finding a balance between kidney function, periprocedural morbidity, the risk of the operation and tumor control was underlined in this study.

Kawada et al. investigated the oncological and safety outcomes, comparing endoscopic management versus a radical nephroureterectomy [22]. The authors observed no significant differences between the two approaches for low-risk UTUC in terms of overall survival, cancer-specific survival or bladder recurrence-free survival while preserving renal function. Nevertheless, the endoscopic approach was characterized by a higher local recurrence rate that demands rigorous follow-ups and more interventions. The findings of Kawada et al. are supported by Shenhar et al. who found that endoscopic management may offer a possibility of 83% for the preservation of the kidney but is associated with 6.5 ± 4.4 surgeries during a mean surveillance of 4.9 ± 3.4 years. Recent studies have shown that approximately 20–30% of patients treated with endoscopic ablation may develop disease progression and may need to undergo a salvage nephroureterectomy [11]. In our study, one patient underwent three operations before achieving a cancer-free status. Wen et al. presented a retrospective single-center study that revealed that 7 out of 32 patients treated with endoscopic thulium laser ablation for UTUC were diagnosed with tumor recurrence during the follow-up period with a ureteroscopy every three months for a year and every six months since [23]. Three of them underwent a nephroureterectomy. As for post-endoscopic complications, four patients (12.5%) developed a ureteral stricture managed either with balloon dilatation, stenting or even laser incision. This study underlines the complexity of a therapeutic approach for UTUC patients and the importance of a thorough discussion regarding the treatment that should be conducted before a common decision. In our study, one patient presented a stricture of the ureteropelvic junction after the percutaneous resection of the tumor. Additionally, in their systematic review, Laukhtina E et al. [24] reviewed the outcomes between the retrograde and antegrade approach for patients with UTUC. The bladder recurrence rate was 35% after a retrograde endoscopic surgery, while it was found to be 17.7% after an antegrade endoscopic surgery. The upper urinary tract recurrence rate was 56.4% and 36.2%, respectively. The study demonstrated the favorable safety profile of the retrograde endoscopic approach for UTUC, as well as the low incidence of postoperative ureteral strictures or major complications. Nevertheless, the high recurrence rate in the upper urinary tract following this procedure may highlight the limitations of current ureteroscopic tools for tumor visualization, resection and extraction. The bibliography suggests that 22–47% of patients with UTUC will present with bladder recurrence, while 2–6% will present with recurrence in the contralateral upper urinary tract [25]. Synchronous upper urinary tract disease is very rare (1.6%) [26]. Previous surgical exposure and instrumentation might play an important role in the metachronous presentation of the disease in terms of risk increase, as it underlined by Sountoulides et al. [27]. The authors revealed that 7.2% of the patients that were treated for bladder cancer with a double j-stent placement presented metachronous UTUC, while the percentage of patients in the same situation after a nephrostomy placement was 2.3%. In the current study, two of the patients presented with metachronous bladder cancer, followed by metachronous UTUC. Nevertheless, only the necessary instrumentation for the diagnosis and treatment of the disease was conducted.

A segmental ureterectomy is a therapeutic option mainly preserved for tumors located mostly in the distal ureter and presents comparable oncological outcomes to a radical nephroureterectomy in terms of cancer-specific and recurrence-free survival rates [28]. A distal ureterectomy and ureteroneocystostomy with or without a Boari flap/psoas hitch prevail in endoscopic ablation in terms of the tumor’s pathological stage and grade. A distal ureterectomy followed by a ureteroneocystostomy, with or without a psoas hitch or Boari flap, is the most frequent form of segmental resection [11]. This approach is recommended for low-risk and selective high-risk UTUC tumors of the distal ureter. One of the advantages of segmental ectomy compared to endoscopic treatment is that it allows for accurate pathological staging and grading while preserving ipsilateral renal function [11]. In their studies, Veccia et al. and Fang et al. presented a comparison between a segmental ureterectomy as a treatment of choice and an RNU, resulting in no significant difference in terms of cancer-specific survival, disease progression or metastases [29,30]. In addition, a recent study by Paciotti et al. comparing a segmental ureterectomy and a radical nephroureterectomy as treatments for UTUC, with a median follow-up of four years, showed that a segmental ureterectomy is not inferior regarding oncological outcomes and overall survival [31]. The necessity of preserving renal function has played a role in the advancement of different techniques that include the use of ileal segments as a substitution for the resected ureteral tissue, while a segmental ureterectomy has also been used for patients with high-risk UTUC [32,33].

Bacillus Calmette-Guerin (BCG) and mitomycin C have been used as an adjuvant treatments to endoscopic ablation of UTUC. There are some promising data presenting mitomycin C gel instillation in the upper urinary tract as a solid choice of treatment, even for high-risk UTUC, without compromising the cancer-free rates [34]. In particular, recent studies have shown that half of the patients with imperative indications treated with mitomycin gel instillations for high-risk UTUC had no evidence of recurrence [35]. Nevertheless, longer follow-ups are necessary for the evaluation of these results. The most common adverse effect of this type of treatment is ureteral stenosis, present in almost 40% of the patients in multiple studies [36]. In a case series of eight patients, Rosen et al. examined mitomycin gel using the antegrade approach and reported a significantly low rate of ureteral strictures (one out of eight patients developed an asymptomatic stricture) [37]. These findings were corroborated by Linehan J et al. in the boundaries of a retrospective multicenter study involving 132 patients managed with mitomycin gel for low-grade disease. The authors reported complete response rates of 48% in the retrograde cases and 60% with the antegrade approach. However, Clavien grade 3 ureteral strictures were significantly more common in the retrograde group (32%) compared to the antegrade group (12%) [36]. In the boundaries of these protocols and settings, the psychological impact of multiple ureteroscopies must be taken into consideration. Last, but not least, photodynamic agents, such as padeliporfin are being used in trials as an endoscopic vascular targeting treatment for UTUC lesions with promising results so far [38]. The perioperative administration of immunotherapy seems also to present promising results, as observed by Kolawa et al. [39]. The use of pembrolizumab, atezolizumab and the combination of nivolumab and ipilimumab have been investigated in small patient samples revealing a satisfactory response rate.

In addition to the oncological outcomes, quality of life (QoL) post-treatment is a critical consideration for UTUC patients. A study by van Doeveren et al. demonstrated that patients who underwent a radical surgery, with many receiving intravesical mitomycin C (MMC) instillations, experienced a temporary decline in physical, role and social functioning immediately after surgery. However, these scores returned to pretreatment levels by three months post-surgery. Fatigue, pain and constipation followed a similar recovery pattern, underscoring the temporary nature of postoperative symptoms. Interestingly, emotional functioning improved at one month post-surgery and remained elevated at three months. The study also found that older patients had better social and emotional functioning despite worse physical outcomes, and male patients experienced better emotional well-being, while surgical complications had a negative impact on social functioning [40].

Kidney-sparing surgeries could be acceptable treatment options for low-risk tumors and selective cases of greater burden that are not able to withstand radical surgeries, such as a radical nephroureterectomy. The preservation of renal function and the reduced surgical morbidity compared to radical treatments remain the key benefits of a kidney-sparing treatment. However, proper patient selection is crucial for a successful treatment. Additionally, the risks of recurrence remain a concern after such kidney-sparing approaches. Further research is required for the optimization of the treatment of high-grade UTUC in patients with solitary kidneys and to confirm the long-term efficacy of the current kidney-sparing options in this population.

In the current study, we presented three cases with solitary kidneys and UTUC, a situation that makes renal preservation imperative. Our results suggest that kidney-sparing management could be a viable option for patients with solitary kidneys, even in the case of high-grade UTUC. The patients remain without metastases; nevertheless, these findings should be interpreted within the context of their limitations. First of all, the number of patients is restricted and should not result in absolute conclusions. In addition, the duration of the follow-ups applies to the aforementioned restrictions. Despite its limitations, this study presents a small sample of patients who have been successfully managed, following a strict surveillance schedule. A greater sample of patients and a longer follow-up period may strengthen the outcomes of this retrospective study.

## 5. Conclusions

The kidney-sparing approach (tumor laser ablation or resection) for high-risk UTUC treatment in the selected patients with solitary kidneys presents acceptable early outcomes regarding renal function preservation and disease management. A strict follow-up is demanded, and it must be respected not only by the patient but by the physicians as well. Any pathological result may demand further diagnostic investigations and operations. It is important to personalize the choice of treatment in every case after a thorough examination of the patient’s data.

## Data Availability

The data that support the findings of this study are available from the corresponding author, upon reasonable request.
